# STICKS study – Short-sTretch Inelastic Compression bandage in Knee Swelling following total knee arthroplasty – a feasibility study

**DOI:** 10.1186/s13063-016-1767-5

**Published:** 2017-01-09

**Authors:** T. M. Brock, A. P. Sprowson, S. Muller, M. R. Reed

**Affiliations:** 1 Trauma and Orthopaedic Surgery, Newcastle University, Newcastle upon Tyne, NE1 7RU UK; 2 Trauma and Orthopaedics, Warwick Clinical Trials Unit, University of Warwick, Coventry, CV4 7AL UK; 3 Wansbeck General Hospital, Northumbria Healthcare NHS Foundation Trust, Woodhorn Lane, Ashington, NE63 9JJ UK

**Keywords:** Knee replacement, Arthroplasty, Compression bandage, Enhanced recovery, Fast track, Feasibility

## Abstract

**Background:**

Postoperative knee swelling is common and impairs early postoperative function following total knee arthroplasty. It was hypothesised that the use of a short-stretch, inelastic compression bandage would reduce knee swelling and improve pain and early function. The aim of this study was to provide preliminary data and test feasibility with a view to informing a larger, future trial.

**Methods:**

Fifty consecutive patients selected for primary total knee arthroplasty underwent distance randomisation to receive a short-stretch, inelastic compression bandage or a standard wool and crepe bandage for the first 24 h postoperatively. Study feasibility including recruitment rates, retention rates and complications were analysed. The Oxford Knee Score, the EQ-5D-3L index score, knee swelling, knee range of motion, visual analogue pain score and length of stay were compared between groups. Analysis of covariance (ANCOVA) was performed adjusting for the preoperative measurement.

**Results:**

Sixty-eight percent of eligible patients were recruited into the trial. The retention rate was 88%. There were no complications regarding compression bandage use. There was a greater mean but non-significant improvement in Oxford Knee Score (*p* = 0.580; point estimate = 2.1; 95% CI −3.288 to 7.449) and EQ-5D-3L index score (*p* = 0.057; point estimate = 0.147; 95% CI −0.328 to 0.005) in the compression bandage group at 6 months. There was no significant difference between groups regarding knee swelling, knee range of motion, visual analogue pain score, complications and length of stay.

**Conclusion:**

Preliminary data suggests that the use of an inelastic, short-stretch compression bandage following total knee arthroplasty is a safe technique that is acceptable to patients. A larger, multicentre trial is required to determine its effect postoperatively.

**Trial registration:**

The study was registered with Current Controlled Trials, identifier: ISRCTN86903140. Registered on 30 May 2013.

## Background

Total knee arthroplasty is a common and highly successful operation in the management of osteoarthritis. However, postoperative knee swelling is a common problem due to intra-articular bleeding and inflammation of periarticular tissues [1]. This results in decreased functional performance as a result of quadriceps weakness [2] and arthrogenic reflex inhibition due to pain [3] which can delay rehabilitation, increase hospital length of stay and decrease patient-reported outcomes [4, 5]. Additionally, excessive knee swelling is associated with increased rates of wound dehiscence and infection [6].

Intraoperative techniques to reduce intra-articular bleeding, such as surgical technique [7], tourniquets [8] and medication [9] are features in enhanced recovery programmes. However, postoperative methods, including the use of a cold compress [10], cryotherapy [11], elastic bandaging [12] and compression bandages [13, 14] have had limited success.

Compression bandage therapy is the established treatment of venous ulcers and lymphoedema [15, 16]. It is hypothesised that the application of this external compression aids venous return and reduces hydrostatic pressure in the leg by (1) improving the efficacy of the calf-muscle pump and (2) moving blood from the superficial to the deep venous system, subsequently allowing movement of fluid from the interstitial space. The use of inelastic bandages are preferred in arthroplasty as they have a low, tolerable resting pressure but a more effective activation of the deep venous system and calf-muscle pump with ambulation compared to their elastic counterparts [17].

The efficacy in total knee arthroplasty is still unclear due to conflicting results in the medical literature and heterogeneous methodology [13, 18–20]. We hypothesised that the use of a compression bandage in total knee replacement would improve postoperative pain, swelling and functional outcomes. The aim of this study was (1) to test study feasibility in the form of eligibility, recruitment rate, attrition rates and bandage complications, (2) to derive preliminary subjective and objective data and (3) to derive a power calculation for the Oxford Knee Score using preliminary data.

## Methods

**Fig. 1:**
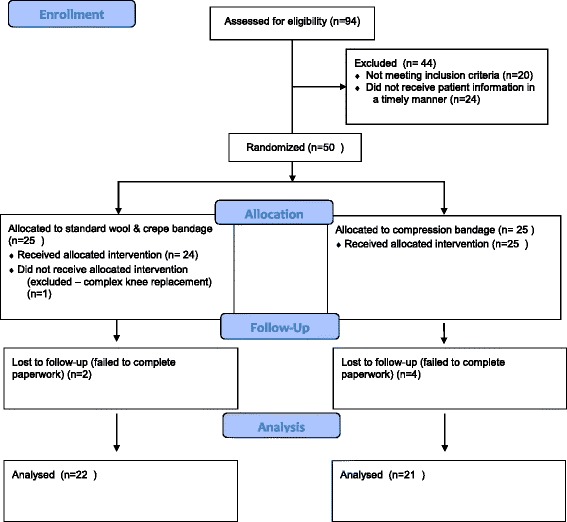
Consolidated Standards of Reporting Trials (CONSORT) flow chart

The study was a prospectively enrolled, randomised controlled feasibility study conducted at two hospital sites within Northumbria Healthcare NHS Foundation Trust. Ethical approval was obtained (13/NE/0137) and the study was registered with Current Controlled Trials (ISRCTN86903140). A protocol paper with complete methodology was published previously [21]. A Consolidated Standards of Reporting Trials (CONSORT) flow chart (Fig. 1) was utilised.

### Participant recruitment

Fifty patients selected for total knee arthroplasty were enrolled in the study between November 2013 and June 2014. The inclusion criteria for the study included (1) primary total knee arthroplasty for osteoarthritis, (2) age over 18 years and (3) being able to provide written, informed consent. Exclusion criteria included (1) peripheral vascular disease characterised by an Ankle-Brachial Pressure Index (ABPI) <0.8, (2) peripheral neuropathy and (3) Body Mass Index (BMI) >40.

Patients were randomised on the day of surgery by electronic distance randomisation using the website http://www.sealedenvelope.com. The randomisation process was done in random permuted blocks to allow even balancing of the groups. Patients were allocated to the control group or the compression bandage group. There was no difference in baseline characteristics (Table 1). Preoperative measurements (below) were recorded prior to randomisation to reduce bias.

**Table 1:** Patient demographics. Standard deviations in parentheses

	Control (*n* = 25)	Compression (*n* = 24)
Age	69.5 (6.8)	67.3 (8.2)
Female gender	16	16
Body Mass Index	28.8 (4.4)	29.7 (5.5)
Oxford Knee Score	23.3 (7.9)	22.7 (8.3)
EQ-5D-3 L index score	0.554 (0.270)	0.570 (0.240)
Knee ROM (degrees)	93.0 (28.4)	103.7 (15.7)
Calf circumference (cm)	37.3 (4.3)	37.8 (3.2)
Knee girth (cm)	41.7 (4.1)	41.8 (3.9)
Thigh girth (cm)	46.0 (6.0)	46.9 (4.5)
Visual analogue pain score	4.8 (2.4)	4.6 (2.7)

*ROM* range of movement

### Control group

Patients underwent primary total knee arthroplasty under the care of one of two consultant orthopaedic surgeons (SM, MR). Surgery was performed under general anaesthesia or spinal anaesthesia and sedation. Intra-venously administered (IV) antibiotics (gentamicin 3 mg/kg and teicoplanin 400 mg IV) and tranexamic acid (30 mg/kg IV up to 2.5 g) were administered and a tourniquet used. A Nexgen cruciate-retaining total knee arthroplasty was used (Zimmer, Swindon, United Kingdom) with Palacos R + G bone cement (Heraeus Medical, Newbury, United Kingdom). Intra-operative periarticular injections of 80 ml 0.125% bupivacaine were infiltrated and a further 20-ml 0.125% bupivacaine bolus given via intra-articular wound catheter after wound closure. The skin was closed using surgical skin clips, which were removed at 10 to 14 days post-operatively. A hydrocolloid dressing (Aquacel Surgical, Convatec Ltd., Flintfield, UK) was used for the wound. Standard bandaging consists of a soft inner layer (Soffban, BSN Medical Ltd., Brierfield, UK) applied from 10 cm below to 10 cm above the patella with a 50% overlap of bandage, followed by a similar outer layer of crepe bandage (BSN Medical Ltd., Brierfield, UK) prior to deflation of the tourniquet.

The bandage and wound catheter were removed at 24 h leaving the hydrocolloid wound dressing in situ. This dressing stayed on until the clips were removed at 10–14 days. A cryocuff was used after 24 h.

### Compression bandage group

Patients received a compression bandage over the hydrocolloid surgical wound dressing instead of the routine wool and crepe bandage. Following tourniquet removal a soft inner layer (Soffban, BSN Medical Ltd., Brierfield, UK) was applied from the toes to the groin on the affected leg with a 50% overlap of bandage. Following this the outer compressive layer bandage (Actico bandage, Activa Healthcare Ltd., UK) was applied firmly over the top, again with a 50% overlap of bandage. The bandage was pulled to full stretch before it was wrapped around the leg to ensure adequate compression in the application. It was applied after release of the tourniquet by necessity due to its length up the thigh. To ensure homogeneity in bandage application, the operating surgeons were shown a training video on correct application of the bandage and were given a tutorial on bandage application with real-life bandage application and feedback. The bandage was removed at 24 h post surgery leaving the hydrocolloid wound dressing in situ.

### Study feasibility

Recruitment, retention and refusal rates were calculated. The proportion of patients undergoing primary total knee arthroplasty who did not meet the eligibility criteria was also calculated to ensure this was not too restrictive. Ease of application and tolerance of the compression bandage was determined qualitatively by the operating surgeons and patients, respectively. Potential adverse risks associated with the use of the compression bandage, such as pain, wound dehiscence and blistering, were recorded and rates calculated.

### Outcome measures

The Oxford Knee Score was measured preoperatively and at 6 months in line with UK Health and Social Care Programme Patient Reported Outcome Measures. The EuroQol EQ-5D-3L questionnaire was measured preoperatively and at 6 months.

Knee swelling and range of motion were measured preoperatively, daily until discharge and at 6 weeks. Visual analogue pain scores were recorded preoperatively, daily until discharge (pre- and post-physiotherapy) and at 6 weeks.

Length of hospital stay, readmission rates and complications were also recorded using hospital episode statistic data.

### Statistical analysis

A secure Excel database was used to record data (Microsoft Inc., Albuquerque, NM, USA). Statistical analysis was performed using SPSS version 23.0 (SPSS Inc., Chicago, IL, USA).

For each outcome measure (Oxford Knee Score, EQ-5D-3L index score, knee swelling, range of motion, visual analogue scale score) the two groups were compared at each applicable set time point (preoperative, day 1, day 2, 6 weeks, 6 months) using analysis of covariance (ANCOVA) with the baseline preoperative measurement set as the co-variate. Statistical significance was denoted at *p* < 0.05.

A power calculation was performed based on the mean and standard deviations of the Oxford Knee Scores collected from this data to inform a future, larger trial. The risk of a type 1 error was set at 0.05 and a type II error at 0.20. The retention rate from feasibility data was incorporated into the final sample size number to allow for possible patient dropout.

## Results

### Study feasibility

Of the 94 total knee arthroplasties performed during the time period, 20 (21%) did not meet the inclusion criteria. Using the 74 eligible patients, the recruitment rate was 68% (*n* = 50). Thirty-two percent (*n* = 24) of patients were seen in clinic but did not receive patient information in a timely manner and were not enrolled. There were no refusals of participation in the study.

During the trial, one patient was excluded from the trial in the control group due to an unplanned complex knee replacement which did not meet the inclusion criteria. Two patients in the control group and four patients in the compression bandage group did not complete 6-month follow-up and could not be analysed (dropout rate 12%) (Fig. 1).

There were no reported problems with bandage application by the operating surgeons and the bandage was well-tolerated by patients, with no reports of discomfort or restriction. There were no reported skin complications with the compression bandages.

### Outcomes

1. *Oxford Knee Score*
  There was a mean Oxford Knee Score improvement of 11.0 in the control group and 13.1 in the compression bandage group. Whilst the point estimate (2.1) was nearing the minimally clinically significant difference of 3.0, the change in score was not statistically significant (*p* = 0.580; 95% CI −3.288 to 7.449) (Table 2)

**Table 2:** Oxford Knee Score and EQ-5D-3L index measurements – standard deviations in parentheses. Point estimate refers to mean difference between groups

	Control (*n* = 25)	Compression (*n* = 24)	Point estimate	*p* value	95% CIs
Pre-op EQ-5D-3L index	0.554 (0.270)	0.570 (0.240)	0.016	-	-
6-month EQ-5D-3L index	0.651 (0.331)	0.812 (0.183)	0.163	0.057	−0.328 to 0.005
Pre-op Oxford score	23.3 (7.9)	22.7 (8.3)	−0.6	-	-
6-month Oxford score	34.3 (10.6)	35.8 (7.7)	1.5	0.580	−3.288 to 7.449
2. *EQ-5D-3L index*
  There was a mean improvement in the EQ-5D-3L index score in the compression bandage group compared to the control group at 6 months, but this was not statistically significant (*p* = 0.057; point estimate = 0.147; 95% CI −0.328 to 0.005) (Table 2)
3. *Knee swelling*
  There was an increase in mean knee swelling in both groups on day 1 post-operatively, which increased further at day 2 post-operatively. However, these values were close to pre-operative levels by week 6, with the exception of the knee circumference in both groups. There was, however, no significant statistical difference between groups at any time point (Fig. 2)

**Fig. 2:**
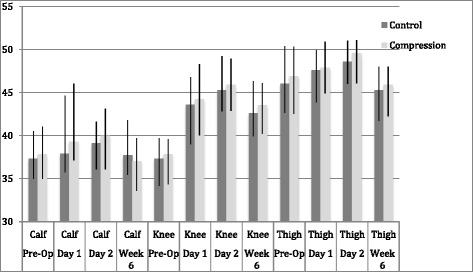
Bar chart to show mean circumference (cm) of the leg pre- and post-total knee arthroplasty. Error bars represent interquartile range. No statistical significance was found between groups at any time point
4. *Range of motion*
  There was a decline in mean range of motion post-operatively in both groups, which decreased further at day 2 post-operatively. However, similarly to knee swelling, these values were close to pre-operative levels by week 6. There was large variation in initial measurements in both groups, reflecting the varying degrees of restriction of the knee due to osteoarthritis and fixed-flexion deformity. There was no significant statistical difference between groups at any time point (Fig. 3)

**Fig. 3:**
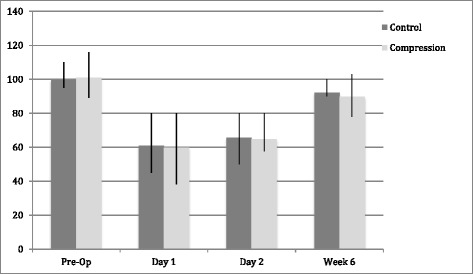
Bar chart to show mean total range of motion (degrees) of the leg pre- and post-total knee arthroplasty. Error bars represent inter-quartile range. No statistical significance was found between groups at any time point
5. *Pain scores*
  There was an improvement between pain scores in both groups at 6 weeks compared to pre-operatively. There was a small increase in pain scores in the compression bandage group on day 1 and day 2 but this was not significant. There was no significant statistical difference in pain scores between groups at any time point (Table 3)

**Table 3:** Mean visual analogue pain score – standard deviations in parentheses. Point estimate refers to mean difference between groups

	Control (*n* = 25)	Compression (*n* = 24)	Point estimate	*p* value	95% CIs
Pre-op	4.8 (2.4)	4.6 (2.7)	−0.2	-	-
Day 1 pre physio	3.9 (2.7)	4.5 (2.7)	0.6	0.974	−1.660 to 1.685
Day 1 post physio	5.1 (2.6)	6.0 (2.6)	0.9	0.307	−0.802 to 2.376
Day 2 pre physio	2.4 (2.3)	4.2 (2.3)	1.8	0.067	−0.180 to 2.625
Day 2 post physio	4.5 (2.3)	5.2 (2.7)	0.7	0.726	−1.144 to 1.829
Week 6	2.6 (1.6)	2.8 (2.2)	0.2	0.244	−0.560 to 2.110
6. *Length of stay and complications*
  The mean length of stay was 3.3 days in the control group and 3.1 days in the compression group (*p* = 0.749; point estimate = 0.2; 95% CI −0.815 to 1.125). There were no registered infections or thromboembolic events in either group

## Discussion

The main finding of this feasibility study was that the study was acceptable to patients with 68% recruitment rate of eligible patients. Attrition was relatively low at 12% and the intervention was acceptable to patients with no documented complications associated with the bandage use.

In terms of clinical outcomes there was a greater mean improvement in the Oxford Knee Score in the compression bandage group. Whilst the 95% confidence interval (−3.288 to 7.449) was wide it did include the three-point difference associated with a clinically important difference. There was a mean greater mean improvement in EQ-5D-3L index scores at 6 months in the compression bandage group, but this was not statistically significant (*p* = 0.057; point estimate = 0.147; 95% CI −0.328 to 0.005). No statistical difference was found between the groups regarding knee range of motion, knee swelling or pain at any time point.

We accept that there are limitations with the current study. With regards to the study protocol, it was not possible to blind patients or investigators to the intervention, which may introduce bias. Secondly, interobserver variation is a known limitation of objective knee measurements including range of motion and circumference. It is possible that this may account for some of the variation between subjects and consequently, in combination with the large sample size required for the definitive trial, will not be used. Thirdly, there was a large proportion of patients who were eligible for the trial but not enrolled (32%). This was due to the logistics of research nurse availability and not patient demographics or comorbidities. All patients who were approached regarding the trial agreed to participate. Finally, whilst the primary aim was to ascertain feasibility of the study, the study measurements were underpowered and at risk of type II error. Using this preliminary data for Oxford Knee Score, incorporating attrition rate and standard deviation with 90% power, we would need the following number of participants to determine statistical significance: 354; 790; 3088 for a 3-, 2- and 1-point difference, respectively. These figures will form the basis of the future multicentre study.

In a randomised controlled trial of 60 patients undergoing unicondylar knee arthroplasty, Pinsornsak et al. found no difference in swelling, blood loss or pain at 24 h and 48 h postoperatively between a modified Robert-Jones bandage and a standard wool and crepe bandage [19]. Whilst the bandaging technique was different, the findings are similar to our study.

In contrast, Charalambides et al. found that patients had improved range of motion (knee flexion) and decreased length of stay when a compression bandage was used [14]. However, the study of 150 patients was not randomised and relied on retrospective data in the cohort that received the standard wool and crepe bandages. It was also not performed in an enhanced recovery programme setting. A study by Cheung et al. found similar findings regarding improved knee flexion in the compression bandage group, with associated lower incidence of walking aids at discharge [20]. Anderson et al. found that the use of a compression bandage and local anaesthetic infiltration was associated with significant improvement in pain scores at 8 h but not 24 h postoperatively [13]. In our present study, no significant difference in pain scores were seen 24 h postoperatively. The local anaesthetic infiltration in both our groups may have influenced this. There was a small, non-significant increase in pain scores in the compression bandage group during day 1 and day 2 post-operatively, but this did not tally with qualitative information on the bandage provided by the patients.

To our knowledge this is the first study to utilise patient-reported outcome measures with the use of compression bandages after total knee arthroplasty. Whilst not statistically significant, the mean improvement in Oxford Knee Score of 2.1 in the compression bandage group is near the minimal clinically significant difference for the Oxford Knee Score [22]. The greater mean improvement in the EQ-5D-3L index score (*p* = 0.057; point estimate = 0.147; 95% CI −0.328 to 0.005) in the compression group also follows this trend. However, these results should be interpreted with caution due to the small sample size. As the compression bandage was easy to apply and well-tolerated by patients, without any documented complications, further investigation of its use in a larger, multicentre trial is warranted.

## Conclusion

Feasibility data suggests that the compression bandage is easy to use and well-tolerated. No statistically significant differences were seen for post-operative scores between groups. However, there was a marginal trend to an improvement in patient-reported outcome measures at 6 months in the compression bandage group. Future work should be directed at further investigation of these patient-reported outcome measures in a larger, multicentre trial.
